# Revealing the Mechanisms for Linalool Antifungal Activity against *Fusarium oxysporum* and Its Efficient Control of Fusarium Wilt in Tomato Plants

**DOI:** 10.3390/ijms24010458

**Published:** 2022-12-27

**Authors:** Xiuming Li, Qifang Wang, Haosen Li, Xiaoyun Wang, Ruimin Zhang, Xiaoyu Yang, Qiwei Jiang, Qinghua Shi

**Affiliations:** College of Horticultural Science and Engineering, Shandong Agricultural University, Tai’an 271018, China

**Keywords:** volatile compounds, antifungal mechanism, *Fusarium oxysporum f. *sp. *radicis-lycopersici*, Fusarium crown and root rot, transcriptomic and proteomic

## Abstract

*Fusarium oxysporum f. *sp.* radicis-lycopersici* (*Forl*) is a destructive soil-borne phytopathogenic fungus that causes Fusarium crown and root rot (FCRR) of tomato, leading to considerable field yield losses. In this study, we explored the antifungal capability of linalool, a natural plant volatile organic component, against *Forl* and its role in controlling FCRR symptoms in tomatoes. Our results showed that *Forl* mycelial growth was inhibited by the linalool treatment and that the linalool treatment damaged cell membrane integrity, enhanced reactive oxygen species levels, depleted glutathione, and reduced the activities of many antioxidant enzymes in *Forl*. Transcriptomic and proteomic analyses demonstrated that linalool also downregulated metabolic biosynthetic pathways at the transcript and protein levels, including redox, transporter activity, and carbohydrate metabolism in *Forl*. Moreover, linalool significantly decreased the expression of many *Forl* pathogenic genes, such as cell wall degrading enzymes (CWDEs) and G proteins, which is likely how a *Forl* infection was prevented. Importantly, exogenously applied linalool activated the salicylic acid (SA) and jasmonic acid (JA) defensive pathways to improve disease resistance and relieved the negative effects of *Forl* on plant growth. Taken together, we report that linalool is an effective fungicide against *Forl* and will be a promising green chemical agent for controlling FCRR.

## 1. Introduction

*Fusarium oxysporum f. sp. radicis-lycopersici* (*Forl*) is a soil-borne fungus pathogen that causes Fusarium crown and root rot (FCRR) symptoms in tomatoes and many other hosts, leading to considerable field and greenhouse yield losses [1]. *Forl* invades from the root hair zone and moves acropetally through the xylem, where it blocks the vessels after producing enzymes and toxins, resulting in yellowing, wilting, and the death of the plant [1]. The stem vascular tissue browns in infected plants [2]. *Forl* can survive in soil for a very long time; once introduced into a field, it is almost impossible to eliminate, thus, making it extremely difficult to control [2]. *Forl* remains a major threat to tomato production [3]. Chemical fungicides and soil fumigants were extensively applied for a long time to control *Forl* during the commercial production of tomatoes. However, they are of limited usefulness during a disease outbreak, are hazardous to the environment, and have some negative impacts on food safety [4]. Therefore, natural plant products, such as plant extracts, volatile organic compounds (VOCs), and resins, are continuously being developed as alternatives to fungicides for the management of *Forl* [5,6,7].

Plants normally produce VOCs and diffuse into the atmosphere during different developmental phases or when the plant is attacked by biotic or abiotic stressors [8,9]. These VOCs are a ubiquitous chemical communicating system between the environment and surrounding organisms [9,10]. Plants use VOCs to attract pollinators for pollination, repel herbivores, and protect themselves from pathogenic attacks [8,9,11]. In addition, VOCs mitigate abiotic stressors and consequently increase plant fitness [12]. Recently, the antimicrobial activities of VOCs (e.g., antibacterial and antifungal) have attracted attention as a target to improve agricultural production safety and quality [13].

Linalool (3,7-dimethyl-1,6-octadien-3-ol) is a common multifunctional volatile monoterpene alcohol detected in plants [14]. Due to its unique aroma and flavor, as well as antimicrobial, anti-inflammatory, anti-anxiety, and antioxidant activities, linalool is commonly used in the cosmetics, food, and pharmaceutical industries [15]. When stimulated by pathogens [16], herbivore bites [17,18], plant defense relative hormones JA [19,20] and SA [21], plants emit more linalool. Many studies have shown that linalool inhibits a wide spectrum of phytopathogenic bacteria and fungi in vitro [22]. For examples, linalool has strong antibacterial activity against the foodborne bacteria *Pseudomonas fluorescens* [23], *Salmonella typhimurium* [24], and *Listeria monocytogenes* [25], by disrupting the cell membranes or inactivating biofilm formation on the surface of food. In addition, linalool has strong antifungal activity against the strawberry gray mold fungus *Botrytis cinerea* by downregulating ergosterol in the fungal cell membrane, impairing membrane integrity, damaging the mitochondrial membranes, and decreasing ATP content [16]. Linalool also inhibits pathogenicity by regulating plant defense responses and frequently interacts with other hormonal signaling pathways in vivo. The exogenous application of JA upregulates the expression of the *linalool synthase* (*LIS*) gene and promotes the accumulation of linalool in tomatoes and rice [19,20]. Transgenic rice plants overexpressing *OsLIS* accumulate higher levels of linalool and have significant antibacterial activity against *Xanthomonas oryzae* pv. *oryzae* (*Xoo*). Vapor treatment with linalool also enhances resistance to *Xoo* in rice via inducing the expression of defense-related genes [20]. Linalool exhibits strong antibacterial and antifungal activities against the citrus canker bacterium *Xanthomonas citri* subsp. *citri* (*XCC*) and the fruit decay fungus *Penicillium italicum* (*PI*) in vitro [26]. Moreover, the accumulation of linalool is much higher in resistant species (Ponkan mandarin) than in susceptible species (such as Eureka lemon and Mato Buntan pummelo) in most tissues, suggesting that linalool biosynthesis and accumulation might be involved in plant defense against bacterial and fungal pathogens [26].

Recently, it was reported that linalool showed directly antibacterial activity against leaf pathogen *Pseudomonas syringae* pv. tomato DC3000 (*Pst* DC3000), which causes bacterial speck disease of tomato, by suppressing the virulence factors in DC3000, thereby significantly reducing the infection and pathogenicity of DC3000 in tomato [27]. Furthermore, linalool could also alleviate postharvest gray mold disease in tomato fruits through regulating the activities of antioxidant enzymes, secondary metabolism enzymes, and cell wall structure related enzymes of tomato fruits [28]. These results indicate that linalool plays an important role in tomato disease control. However, there is no study about the role of linalool in controlling soil-borne fungi, and its effect on *Forl* remains unclear. In this study, the direct inhibiting effect of linalool on *Forl* in vitro was determined, and the ability of linalool to inhibit the infection of tomatoes by the fungus was evaluated. To illuminate the role of linalool in plant–pathogen interactions, we further investigated the key mechanisms of linalool to inhibit the growth of *Forl* and increase resistance against *Forl* at the biochemical, transcriptomic, and proteomic levels.

## 2. Results and Discussion

### 2.1. Linalool Inhibits the Growth of Forl

**Figure 1:**
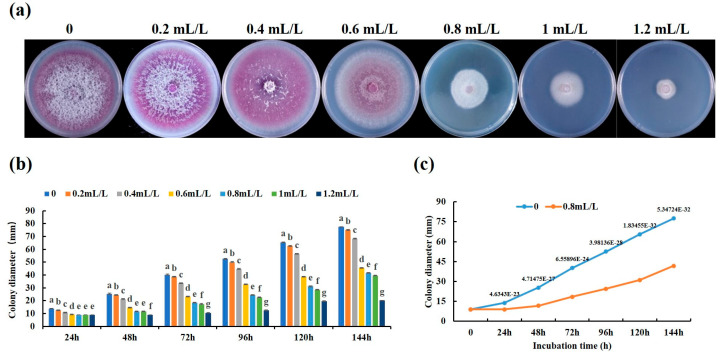
In vitro effect of linalool on *Forl* mycelial growth. (**a**) Mycelial growth of *Forl* on PDA (CK) and PDA supplemented with 0.2–1.2 mL/L of linalool after 6 days of treatment. (**b**) Colony diameters of *Forl* in (**a**). Data are presented as mean ± SD (*n* = 5). Values with different lowercase letters indicate significant differences (Duncan’s test; *p* < 0.05). (**c**) *Forl* colony diameters measured over 6 days (PDA only, blue line, PDA + 0.8 mL/L of linalool, orange line). Each point is the mean of 3 independent replicates (*n* = 5, the value above each point represents *p*-value, Student’s *t*-test).

The effect of linalool on the growth of the *Forl* strain was tested in a sealed Petri dish chamber; as a result, the radial mycelial growth of *Forl* was suppressed by linalool in a dose-dependent manner. As shown in Figure 1a, the mycelial growth of the *Forl* strain was significantly inhibited by linalool at 0.2–1.2 mL/L on PDA medium compared with the control, and the colony color of the mycelia changed gradually from violet to pinkish and eventually to white. After treatment with 0.2, 0.4, 0.8, 1.0, or 1.2 mL/L of linalool for 6 d, the mycelial diameters were 77.491, 75.149, 68.309, 45.396, 41.704, 39.63, and 20.134 mm, respectively, whereas the diameter of the control mycelia was 79.491 mm (Figure 1a,b). These results are expressed as a percentage of inhibition, which was calculated as the ratio of the colony diameters of *Forl* in the control and treatment of linalool, and they are summarized in Table S1. In addition, the EC_50_ values (concentration causing 50% inhibition of mycelial growth) were obtained by fitting the data to a dose–response curve. The results showed that linalool had an antifungal effect on *Forl* in vitro with an EC_50_ value of 0.84 mL/L (Table S1). Therefore, a concentration of 0.8 mL/L was used to assay the effects of linalool on *Forl* growth and disease control in tomato plants. As shown in Figure 1c, 0.8 mL/L of linalool significantly inhibited the growth of *Forl* mycelia 24 h after the treatment and reached a 46% relative growth inhibitory rate 144 h after the treatment. Previous studies have suggested that 1.25 mL/L of linalool is the minimum inhibitory concentration (MIC) for the bacterium *P. fluorescens* [23] and 20.95 μM for the fungus *B. cinerea* [16]. All of these results indicate that linalool has strong antimicrobial activities and that the inhibitory concentration varies among microorganisms.

### 2.2. Transcriptomic and Proteomic Analysis of Forl after the Linalool Treatment

The transcriptome and proteome of *Forl* grown on PDA medium (control) were compared to *Forl* grown on PDA medium supplemented with 0.8 mL/L of linalool for 6 d. A total of 263,369,868 raw reads were obtained from the six samples for the transcriptome analysis. After filtering out the low quality, adapter, and unknown reads, 250,299,230 clean reads remained. Overall, 91.615–97.92% of the clean reads were mapped to the reference genome (*Fusarium oxysporum*, ENSEMBL) (Table S2). A total of 13,974 protein coding genes were detected and annotated by BLAST alignment with six databases (Pfam, PRINTS, ProDom, SMART, ProSite, and PANTHER, Table S3).

**Figure 2:**
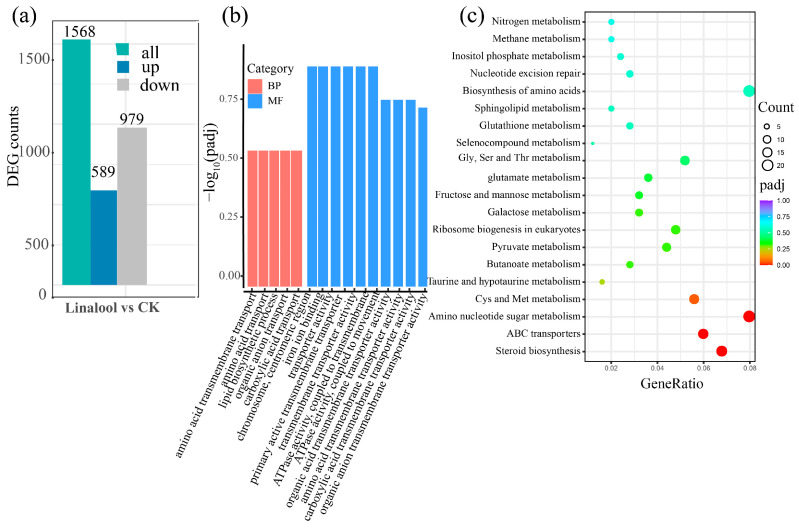
Overview of the data from the transcriptomics analysis. (**a**) DEGs between the CK- and linalool-treated samples. (**b**) GO enrichment analysis of the DEGs. The horizontal axis shows the GO terms in biological processes (BP) and molecular functions (MF). The vertical axis shows the −Log_10_ (*p*-value) value. (**c**) KEGG enrichment analysis of the DEGs. The horizontal axis shows the gene ratio between the number of DEGs and the number of total transcription genes in the corresponding KEGG term. The vertical coordinates indicate the different KEGG terms. The size of the point indicates the gene number in the corresponding KEGG term. The color from purple to red represents the padj value (red represents high significance, while purple represents low).

The gene expression distributions of these six transcriptomes were calculated, and the results illustrated the uniformity of the distribution of expression in the six samples (Figure S1a). The principal component analysis (PCA) revealed significant differences between the control and the linalool-treated samples (Figure S1b). Pearson’s correlation coefficient analysis demonstrated the consistency and satisfactory reproducibility among the biological replicates (Figure S1c). We further conducted a differentially expressed genes (DEGs) analysis using a two-fold change in expression as the cut-off criterion (|log_2_FC| > 1 and *p* < 0.05). A total of 1568 DEGs were obtained after the linalool treatment compared with the control, with 979 differentially upregulated genes and 589 differentially downregulated genes (Figure 2a and Table S4). To elucidate the functions of these DEGs in the *Forl* transcriptome during linalool treatment, Gene Ontology (GO) and Kyoto Encyclopedia of Genes and Genomes (KEGG) enrichment analyses were performed. Based on the GO results, most of the DEGs were categorized into biological processes (BP) and molecular functions (MF), which were mainly enriched in “transmembrane transporter activity”, “ATPase activity”, “lipid biosynthetic process”, “ion binding”, and “plasma membrane” as shown in Figure 2b and Table S5. The top 20 enriched KEGG pathways of the DEGs in response to linalool treatment are shown in Figure 2c. The most enriched KEGG pathways were “steroid biosynthesis”, “ABC transporters”, “amino sugar and nucleotide sugar metabolism”, and “amino acid metabolism”. In addition, some terms associated with oxidation-reduction reactions, such as “pyruvate metabolism” and “glutathione metabolism”, were also detected (Figure 2c and Table S6). 

**Figure 3:**
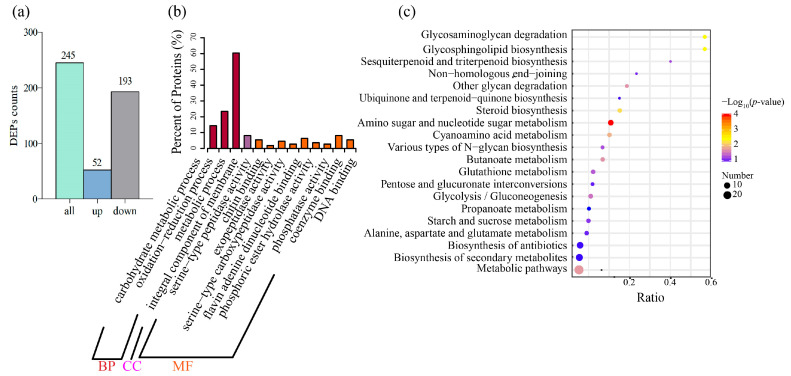
DEPs and the bioinformatics analysis. (**a**) Bar chart of the DEPs between the control and linalool-treated *Forl*. (**b**) GO enrichment analysis of the DEPs. The horizontal axis shows the GO terms, and the vertical axis represents the protein counts ratio among the GO terms. (**c**) KEGG enrichment analysis of the DEPs. The horizontal axis shows the gene ratio between the number of DEPs and the number of total proteins in the corresponding KEGG term. The vertical axis indicates the different KEGG terms. The size of the point indicates the protein number in the corresponding KEGG term. The color from purple to red represents the −Log_10_ (*p*-value) value (red represents high significance, while purple represents low).

To better understand the mechanism of the effect of linalool on *Forl* at the protein level, we analyzed the same treatment using Tandem Mass Tag (TMT)-based quantitative proteomics. A total of 3437 proteins were identified and annotated (Table S7). A total of 245 differentially expressed proteins (DEPs) were identified, of which 52 were upregulated and 193 were downregulated (Figure 3a and Table S8). GO annotation and KEGG enrichment analyses were carried out to gain further insight into their functions and pathways. The details of the GO and KEGG analyses are shown in Table S9 and Table S10, respectively. Based on the GO results, the DEPs were mainly involved in BP terms, such as “carbohydrate metabolism process”, “oxidation-reduction process”, and “metabolism process” (Figure 3b). The DEPs were significantly enriched in the top five pathways, according to the KEGG analysis results, including “amino sugar and nucleotide sugar metabolism”, “glycosaminoglycan degradation”, “glycosphingolipid biosynthesis”, “steroid biosynthesis”, and “cyanoamino acid metabolism” (Figure 3c). Taken together, the GO and KEGG enrichment analyses demonstrated that DEGs and DEPs were associated with “amino sugar and nucleotide sugar metabolism”, “oxidation-reduction process”, “carbohydrate metabolic process”, and “plasma membrane”. Most of the DEPs from these pathways were downregulated, indicating that the antifungal effect of linalool on *Forl* may have resulted from the combined action of plasma membrane perturbation, redox dysfunction, and impeded nutrient metabolism. Therefore, the cell membrane responses, oxidative metabolism, and nutrient metabolic responses to the linalool treatment were given a detailed analysis in the subsequent text.

### 2.3. Linalool Treatment Damages Cell Membrane Integrity

**Figure 4:**
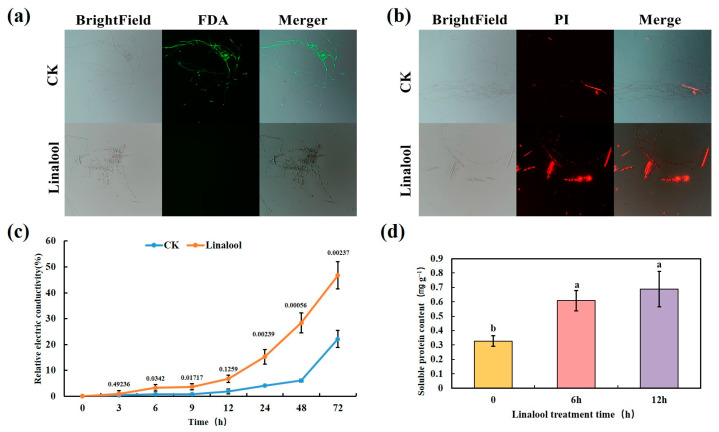
Effect of linalool on cell membrane integrity and cell constituents released by *Forl.* FDA (**a**) and PI (**b**) staining of mycelia and germinated conidia treated with 0.8 mL/L of linalool (bottom), and 0.1% Tween80 (CK, top). Effect of the linalool treatment on electrical conductivity (**c**) and soluble protein contents (**d**) of *Forl*. Columns or points are presented as the average of three independent replicates. The value above each point represents *p*-value (*n* = 3, Student’s *t*-test). Different letters indicate significant differences (Duncan’s test; *p* < 0.05).

The *Forl* activities and cell membrane permeabilization affected by linalool were tested using fluorescein diacetate (FDA) and propidium iodide (PI) staining, respectively. The FDA fluorescent dye stains viable hyphae and produces green fluorescence [27]. The control fungi fluoresced green when stained with FDA. However, *Forl* revealed only faint fluorescence after a 3 h linalool treatment (Figure 4a). Unlike FDA, PI enters dead cells with a disrupted plasma membrane and interacts with the DNA to exhibit red fluorescence [29]. PI fluorescence was observed in the *Forl* linalool-treated hyphae but not in the control samples (Figure 4b). These results demonstrate that the linalool treatment disrupted the cell membranes. Cell membrane integrity is crucial for the maintenance of cell function, as damaging the cell membranes increases permeability and conductivity, and the cell releases its constituents [30]. The electric conductivity of a *Forl* suspension was examined in real time to express the permeability changes in the *Forl* cell membranes. The result showed that the relative electric conductivity (Equation (1)) of the linalool-treated *Forl* suspension was significantly higher (*p* < 0.05) than that of the control after 6 h of linalool treatment and increased rapidly with time (Figure 4c). Cell constituents were also released when examining the soluble protein contents of the *Forl* suspension. Increased leakage of soluble protein was detected after a 6 h linalool treatment compared with the control strain (Figure 4d). All of these results indicate that linalool destroyed the *Forl* cell membrane, increased membrane permeability, and caused cellular leakage. Similarly, the severe damage to the cell membrane structure and membrane permeability caused by linalool has been observed in the bacteria *P. fluorescens* [23], *S. Typhimuriu* [24], and *P. aeruginosa* [31], as well as in the fungi *Candida* [32] and *B. cinerea* [16]. Therefore, the mechanism of action of linalool against bacteria and fungi probably shares some common features.

### 2.4. Linalool Treatment Promotes the Production of Reactive Oxygen Species (ROS) and Inhibits ROS Scavenging in Forl

**Figure 5:**
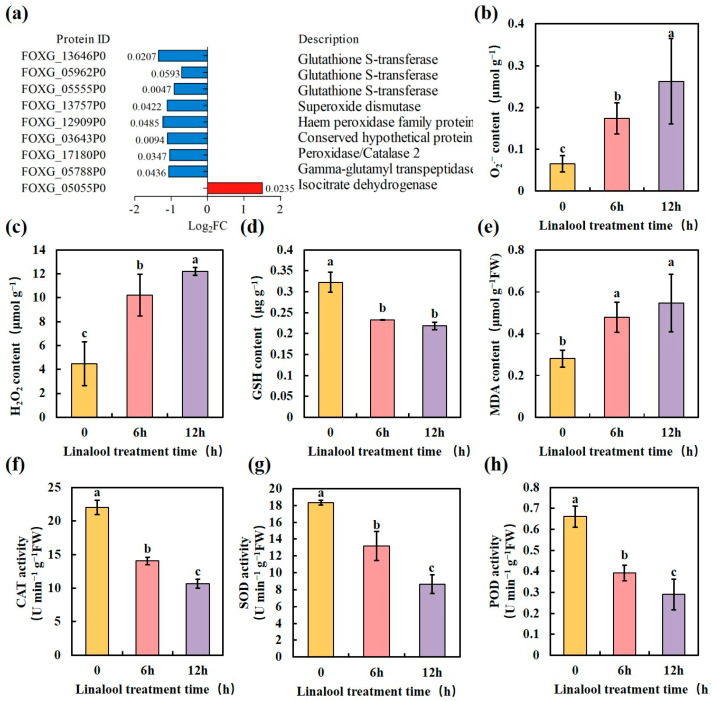
Effect of linalool on the antioxidant metabolism and oxidation level of *Forl*. (**a**) Gene expression of antioxidant enzymes (the values next to the bar represent *p*-values). (**b**, **c**) O_2_•^−^ and H_2_O_2_ contents in *Forl.* (**d**) GSH content. (**e**) MDA content. (**f–h**) ROS scavenging-related enzyme activities: CAT activity (**f**), SOD activity (**g**), and POD activity (**h**). Data are mean ± SD (*n* = 5). Different letters indicate significant differences.

Previous studies have shown that antifungal agents elicit cellular oxidative stress characterized by high ROS content, including superoxide (O_2_•^−^) and hydrogen peroxide (H_2_O_2_) [16,33,34,35]. This massive production of ROS disrupts cellular homeostasis and ultimately leads to cell death. In our proteome data analysis, linalool down-regulated the protein expression of the ROS detoxification enzymes, e.g., glutathione S-transferase (GST), catalase (CAT), superoxide dismutase (SOD), and peroxidase (POD) (Figure 5a), suggesting that the antifungal effect of linalool may result from the redox dysfunction of *Forl*. To further confirm this hypothesis, we determined the ROS contents in linalool-treated *Forl*, and the results showed that the O_2_•^−^ contents (μmol g^−1^) increased about two to three-fold higher than those in the control samples after 6 and 12 h treatments, respectively (Figure 5b). The H_2_O_2_ contents (μmol g^−1^) in the linalool-treated samples increased 2.5 and 4.5-fold compared to those in the control samples after 6 and 12 h treatments, respectively (Figure 5c). Consistent with the increased production in ROS, the glutathione (GSH) content (μg g^−1^) in linalool-treated *Forl* was about 28% lower than that in the control samples after 6 h of treatment (Figure 5d). Adding linalool depleted GSH, leading to increased ROS contents in *Forl* (Figure 5b–d). Lipid peroxidation of cell membranes occurs as a result of excess ROS accumulation, and malondialdehyde (MDA) is a reliable indicator of lipid membrane peroxidation [36]. MDA contents in the control and the linalool-treated *Forl* were compared after the 6 and 12 h treatments. As shown in Figure 5e, more MDA accumulated (Equation (2)) in the linalool-treated *Forl*, suggesting a more severe degree of lipid peroxidation. To better understand the effect of linalool on ROS metabolism, the activities of the antioxidant enzymes CAT, POD, and SOD were measured in *Forl*. After 6 h of treatment, SOD, CAT and POD activities (U min^−1^ g^−1^ FW) in the linalool-treated *Forl* decreased significantly (*p* < 0.05) by 28%, 36%, and 40%, compared to the control samples, respectively, and then significantly decreased even further after 12 h of treatment (Figure 5f–h). These results are consistent with the absolute quantification by the TMT-based proteomic analysis (Figure 5a). The downregulated activities of CAT, SOD, and POD (Figure 5a,f–h), also supported that the antifungal activity of linalool against *Forl* was caused by oxidative damage through massive production of ROS. Taken together, our data strongly suggest that linalool induced high ROS accumulation in *Forl*, which was related to lower antioxidant enzyme activities and lower levels of antioxidant substances. These results are in accordance with reports in the literature that linalool treatment induces oxidative stress in B. cinerea [16] and Klebsiella pneumoniae [37]. Oxidative damage is a common antifungal mechanism of fungicides and plays an important role in the toxicity of many antifungal agents [38,39]. Excess ROS generate lipid peroxidation, leading to DNA, RNA, and protein lesions [34]. Therefore, it was not surprising that most of the DEPs in “amino acid transport and metabolism”, “post-translational modification, protein turnover, chaperones”, and “translation, ribosomal structure and biogenesis, transcription” were down-regulated in linalool-treated *Forl* (Figure S2). 

### 2.5. Linalool Treatment Affects a Variety of Forl Metabolic Reactions 

According to the COG functional categories of the proteomic results, changes were also observed in the levels of proteins involved in “carbohydrate transport and metabolism”, “lipid transport and metabolism”, and “energy production and conversion” (Figure S3a, Table 1 and Table S11). Key enzymes associated with the tricarboxylic acid (TCA) cycle, including isocitrate dehydrogenase, malate/lactate dehydrogenase, FAD/FMN-containing dehydrogenase, and other dehydrogenases, were differentially expressed (Table 1). The set of *Forl* proteins involved in carbohydrate metabolism, such as hexokinase, glucosamine-6-phosphate isomerase, and mannose-6-phosphate isomerase, decreased (Table 1). Hexokinase is a critical glucose utilization enzyme that produces energy and plays central roles in fungal development [40]. This study showed a decrease in carbohydrate metabolism in linalool-treated hyphae. The lipid metabolism was altered, and 7 of 11 enzymes associated with lipid metabolism were significantly downregulated (Table 1). Based on previous results, we speculate that linalool may cause significant damage to the entire metabolic pathway, including carbon metabolism, amino acid metabolism, and lipid metabolism.

### 2.6. Linalool Suppresses the Virulence of Forl and Confers Antifungal Activity against Forl in Tomato Plants

**Figure 6:**
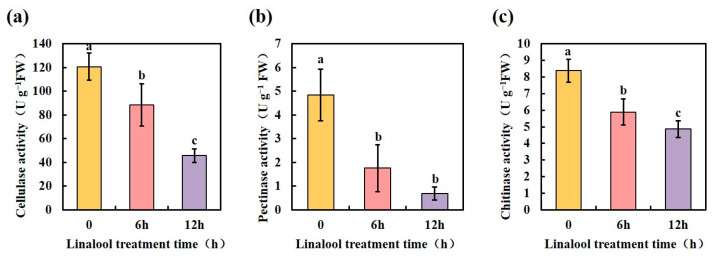
Linalool treatment reduces *Forl* cell wall-degrading enzyme activities. (**a**) Cellulase activity; (**b**) pectinase activity; (**c**) chitinase activity of *Forl* were detected 6 and 12 h after the linalool treatment. Each value is the average of three independent determinations. Different letters indicate significant differences.

As a hemibiotrophic phytopathogen, *Forl* employs a broad range of infection strategies [41]. During infection and colonization, *Forl* secretes hydrolytic enzymes into the apoplastic space, enabling penetration, propagation, and access to nutrition; these enzymes are collectively called cell wall-degrading enzymes (CWDEs) [42]. In *F. oxysporum*, genes that encode CWDEs, such as *chitinase*, *endo-polygalacturonase* (*pg1*), *exo-polygalacturonase* (*pgx4*), *pectate lyase* (*pl1*), *xylanase* (*xly*), and *lipase* (*fgl1*), have been identified [42,43]. The protein annotation results in our proteomic data showed that the glycoside hydrolase family of proteins comprised the majority of the enriched DEP categories (Figure S3b). Among these, the CWDEs associated with the pathogenicity of *Forl* were significantly down-regulated after the linalool treatment (Table 1, proteins marked with two asterisks). We further determined the activities of the CWDEs 6 and 12 h after the linalool treatment. As shown in Figure 6, the enzyme activities of cellulase, pectinase, and chitinase in *Forl* were 27%, 64%, and 29% lower after 6 h of linalool treatment, respectively, compared to the control. The activities of these enzymes decreased more when the treatment was prolonged to 12 h (Figure 6). Other pathogenicity genes that encode Ras proteins (small GTPases) and G-protein signaling components [44] were identified in our proteomic analysis, and their protein expression levels were suppressed in linalool-treated *Forl* (Table 1, proteins marked with one asterisk). The downregulation of CDWE activities and other pathogenicity-related proteins by linalool indicates that linalool may inhibit *Forl* virulence in vivo. 

**Figure 7:**
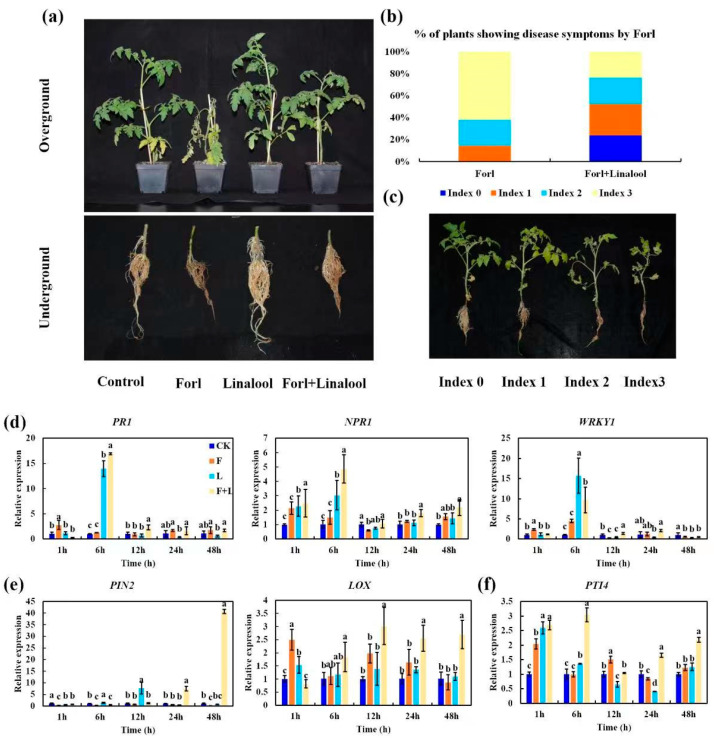
Effect of linalool against *Forl* on tomato plants. (**a**) Symptoms of Fusarium wilt and root rot in aboveground and belowground parts were analyzed and photographed 15 days after inoculation (dpi). (**b**) Plot showing percentage of tomato plants with disease symptom severity represented by the four classes, as shown in the lower panels. (**c**) Disease index of tomato plants. Transcription patterns of the SA-related genes *PR1*, *NPR1*, and *WRKY1* (**d**), JA response genes *PIN2*, and *LOX* (**e**), and ET inducible gene *Pti4* (**f**) were detected 1, 6, 12, 24, and 48 h post *Forl* inoculation. Different letters indicate significant differences.

The potential antifungal activity of linalool against *Forl* in vivo was assessed in tomato plants. The tomato seedlings were subjected to four treatments: (1) control: no fungal inoculation; (2) *Forl*: inoculated with *Forl*; and (3) linalool: treated with linalool; (4) *Forl* + linalool: inoculated with *Forl* and treated with linalool. The infected plants exhibited yellow leaves, wilting, stunting, root rot, and death 15 days after inoculation. In contrast, the tomato plants treated with linalool showed remarkably reduced FCRR symptoms and appeared healthy with more subdued symptoms, as revealed by the appearance of FCRR symptoms (Figure 7a) and evaluated as the disease incidence percentage (%, the number of diseased plants in relation to the total number of tested plants) (Figure 7b,c). The presence of disease symptoms index 3 in the *Forl* + linalool-treated plants decreased from 61.9% to 23.8% compared to *Forl*-inoculated plants. Approximately 24% of the healthy plants (index 0) were observed among the *Forl* + linalool-treated plants, but this did not occur in the *Forl*-treated plants (Figure 7b,c). These observations indicate that linalool controlled the *Forl* infection in tomato plants and could have potential in Fusarium wilt disease management.

Plant defense responses to *Forl* include the activation of pathways dependent on SA, JA, and ethylene (ET) signaling molecules [45]. To confirm the molecular mechanism of linalool-mediated disease resistance, we employed an RT-qPCR to assess the expression patterns of defense-related genes from tomato leaves 1, 6, 12, 24, and 48 h post pathogen inoculation (Figure 7d–f). The expression of the SA response genes *PR1* (*pathogenesis-related gene 1*), NPR1 (*nonexpresser of PR gene 1*), and *WRKY1* [45,46], and the JA response gene *PIN2* (*proteinase inhibitor II*) [47] in linalool-treated plants was robustly induced compared to those of the uninfected control (Figure 7d,e). The ET inducible gene, *PTI4* (*ethylene-responsive factor*) [48], was slightly induced after the linalool treatment, when compared with the uninfected control (Figure 7f). Plants treated with *Forl*+linalool developed a higher expression of *LOX* (*lipoxygenase*, JA synthesis-related gene) [49], *PR1*, *NPR1*, and *PTI4* transcripts than those treated with linalool, *Forl*, and the uninfected control 6 h post inoculation (Figure 7d–f). *PR1* and *NPR1* expression was 16.9 and 4.8-fold higher than that of the uninfected control 6 h after the *Forl*+linalool treatment, respectively (Figure 7d). The *PIN2* expression level in response to the *Forl*+linalool treatment was approximately 7.4-, and 40-fold higher 24 h and 48 h after inoculation, respectively, relative to the non-infected control (Figure 7e). These results suggest that linalool activates the SA and JA-dependent resistance pathways after inoculation with *Forl*. Collectively, we demonstrated that linalool effectively inhibited *Forl* disease development in tomato plants by inhibiting growth, virulence factors, and pathogenicity (Figure 1 and Figure 6 and Table 1), as well as increasing the plants’ defense responses through activation of the SA and JA signaling pathways (Figure 7d,e). This dual function of linalool in pathogen control has also recently been reported in root knot of tomato. Elsharkawy et al. [50] showed the high nematicidal capacity of linalool against root knot nematodes concerning J2 hatching inhibition and mortality. Besides this direct nematicidal activity in vitro, linalool could also induce systemic resistance to root knot nematodes via regulating the expression of defense-related genes (*PR1* and *PAL*) in tomatoes [50]. In tomato and other crops, numerous works have been made in the characterization of linalool in relation to pathogen defense. However, the molecular mechanisms of linalool production and complex genetic regulation under pathogen attack also remain largely unknown. 

### 2.7. Effect of Linalool on Growth of Tomato Plants

**Figure 8:**
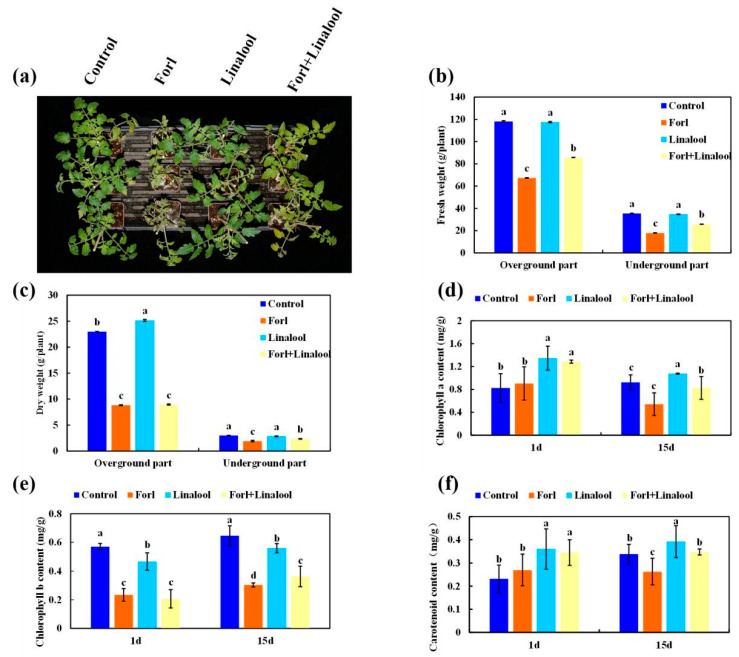
Effect of linalool on growth of tomato plants inoculated with *Forl.* (**a**) Phenotypes of the tomato plants inoculated with water (control), *Forl*, linalool, and *Forl*+linalool at 15 dpi. The tomato plant fresh weight (**b**), dry weight (**c**), chlorophyll *a* content (**d**), chlorophyll *b* content (**e**), and carotenoid content (**f**) were measured at 1 and 15 dpi, respectively. Different letters indicate significant differences.

*F. oxysporum* infection usually reduces photosynthetic activity and generates high levels of intracellular ROS, resulting in cell death and leaf senescence, and consequently, a decline in plant biomass [51]. The morphological traits related to vegetative growth in tomato plants infected with *Forl* deteriorated severely (Figure 7a and Figure 8a). To evaluate whether linalool possessed the ability to overcome this inhibitory effect of *Forl*, the effect of linalool on plant growth was investigated. The fresh weight of aboveground and belowground portions inoculated with *Forl* were significantly lighter by 43% and 49%, respectively, 15 days after inoculation. The reductions in the dry weight of the aboveground and belowground portions were 62% and 36%, respectively (Figure 8b,c). However, a pronounced increase in fresh weight was observed when plants were treated with *Forl*+linalool compared with only *Forl*, and the percentage increases in the fresh weight of the aboveground and belowground portions were 21% and 31%, respectively (Figure 8b). The belowground dry weight increased significantly by 18% in the *Forl*+linalool-treated samples compared with the *Forl*-treated plants, whereas no significant difference was observed in the aboveground dry weight (Figure 8c). One of the most obvious phenotypes of *Forl* infection is yellowing of the leaves, and this process may be directly or indirectly related with leaf chlorophyll content [51]. As shown in Figure 8d,e, the chlorophyll values in the *Forl*-inoculated plants decreased compared with the uninfected control. The percentage decreases in the Chl *a* and Chl *b* contents in the *Forl*-inoculated plants were 40% and 11% compared to the uninfected control, respectively. However, the Chl *a* and Chl *b* contents were higher in the samples treated with *Forl*+linalool compared to those of the *Forl*-inoculated plants after 15 days of treatment, suggesting that linalool alleviated the limits on photosynthesis caused by *Forl* (Figure 8d,e). Similar to the chlorophyll values, the contents of other carotenoid pigments (which act as reducing agents for scavenging ROS during infection) in the *Forl*+linalool-treated plants increased significantly compared to those in the *Forl*-infected plants (Figure 8f). The decrease in the biomass of the plants caused by the *Forl* infection may be related to the reduced photosynthetic activity and oxidative stress mediated by ROS [51,52]. Our results reveal that the promotion of growth by linalool in fungal-infected plants resulted from the combination of enhanced photosynthetic activity and enhanced tolerance to ROS (Figure 8). Several studies have demonstrated that encapsulating linalool—such as oxidized amylose-encapsulated linalool, linalool released from nanofibers, or structurally modifying emulsified linalool droplets—has a greater antimicrobial efficacy than that of pure linalool [22]. Therefore, it is necessary to design a highly efficient, sustained release and stable linalool agent to control significant agricultural pathogens.

## 3. Materials and Methods

### 3.1. Fungal Strain and Growth Conditions

The *Forl* isolate was kindly provided by Prof. Fu Wang of Qingdao Agricultural University [53]. The strain was cultured in potato dextrose agar (PDA) medium for 6 days at 25 °C to obtain conidia. The spore suspension was adjusted to about 1 × 10^7^ spores per mL with sterile distilled water.

### 3.2. Mycelial Growth Inhibition Test

Linalool (98%; Shanghai Aladdin Biochemical Technology Co., Ltd., Shanghai, China) was dissolved with 0.1% Tween80 solution and added to PDA medium to obtain the final linalool solutions of different concentrations (0.2, 0.4, 0.6, 0.8, 1.0, and 1.2 mL/L), and the same volume of 0.1% Tween80 solution was added as the control. *Forl* mycelium agar plugs (9 mm in diameter) from the edge of actively growing *Forl* cultures were placed in the center of the medium and cultured for 6 d. Five replicates were set up for each concentration, and the antifungal effect was determined by measuring the colony diameter using the crossover method. The relative inhibitory rate of mycelial growth, inhibition chance value, the virulence regression equation, and the EC_50_ value were calculated as described by Montenegro et al. [54].

### 3.3. Transcriptome and RNA-seq and Data Analysis

The *Forl* strain was cultured on PDA supplied with 0.8 mL/L of linalool (dissolved in 0.1% Tween80) for 6 d at 25°C. The fungal strain on PDA supplied with only 0.1% Tween80 was cultured as the control. Three biological replicates were established for each treatment. The mycelia were collected to extract RNA using an RNA Extraction Kit (Tiangen Biotech Co., Ltd., Beijing, China). First-strand cDNA was synthesized using random hexamer primer and M-MuLV Reverse Transcriptase, followed by second-strand cDNA synthesis using DNA Polymerase I and RNase H. The cDNA fragments were converted into blunt ends and adaptored with a hairpin loop structure, and these were purified with AMPure XP system (A63880, Beckman Coulter, Beverly, MA, USA). Then, cDNA fragments were performed with a PCR using Phusion High-Fidelity DNA polymerase, Universal PCR primers and Index (X) Primer. The PCR products were purified and were used to generate a library with a TruSeq RNA Library Prep Kit (Illumina, San Diego, CA, USA). Transcriptome sequencing was performed by Tianjin Novogene Bioinformatics Technology Co., Ltd. (Tianjin, China) using an Illumina NovaSeq 6000 system (Illumina, San Diego, CA, USA). After filtering out the low quality, adapter, and unknown reads through in-house Perl scripts, the clean reads were obtained and mapped to the *Fusarium oxysporum* reference genome in the orientation mode using HISAT2 (v2.0.5). In addition, sample repeatability, the distribution of the expressed genes, and a principal component analysis (PCA) were estimated or measured for each sample. The gene expression levels were estimated using the fragments per kilo bases per million mapped reads (FPKM) method. A differentially expressed genes (DEGs) analysis (linalool vs. control) was performed using the DESeq2 R package (1.20.0) [55] and cutoffs of an adjusted *p*-value of 0.05 and |log_2_FC (fold change)| ≥ 1. Gene Ontology (GO) and Kyoto Encyclopedia of Genes and Genomes (KEGG) enrichment analyses were performed to identify the potential functions and pathways of the DEGs using the clusterProfiler R package, and the terms with corrected *p*-values < 0.05 were considered significantly enriched. 

### 3.4. Tandem Mass Tag (TMT)-Based Quantitative Proteomic Analysis 

The *Forl* samples were treated as described in Section 2.3. Protein extraction was performed as described by Gong et al. [56]. Briefly, an appropriate amount of mycelia (100 mg) was taken and ground in liquid nitrogen. The homogenized powder was lysed with 600 μL of lysis buffer (50 mM of Tris buffer, 8 M of urea, 1% SDS, pH = 8), and the protein mixture was reduced with 10 mM of DTT, alkylated with sufficient chloroacetamide, and digested with trypsin overnight at 37 °C. The resulting peptides were loaded on a C18 desalting column and reconstituted in elution buffer (0.1% formic acid and 70% acetonitrile). The eluents of each sample were dried in a spin vacuum and reconstituted in 100 μL of 0.1 M TEAB buffer and 41 μL of TMT Plex labeling reagent (Thermo Fisher Scientific, Inc., Waltham, MA, USA). All labeling samples were mixed in an equal volume, desalted, and separated on a Rigol L3000 high-performance liquid chromatography (HPLC) system. Subsequently, the samples separated by chromatography were subjected to an EASY-nLCTM 1200 UHPLC system (Thermo Fisher Scientific, Inc., Waltham, MA, USA) coupled to an Orbitrap QExactive HF-X mass spectrometer (Thermo Fisher Scientific, Inc., Waltham, MA, USA) operating in data-dependent acquisition mode. 

The resulting raw spectral data were processed with Proteome Discoverer 2.4 (PD 2.4, Thermo Fisher Scientific, Inc., Waltham, MA, USA) and searched against the database (964533-Fusarium_oxysporum.FO2.pep.fasta, 17696 sequences) downloaded from Ensemble Genomes (https://ensemblgenomes.org/). The search parameters were as follows: the mass tolerance for the precursor ion was 10 ppm and the mass tolerance for the product ion was 0.02 Da. The identified peptide spectrum matched at least 1 unique peptide with credibility > 99%, and q-values ≤ 1% of the false discovery rate were retained. Proteins with |Log_2_FC| > 1 values and *p* < 0.05 via the *t*-test were defined as differentially expressed proteins (DEPs).

GO and InterPro functional analyses were conducted using the InterProScan program against the non-redundant protein databases (including Pfam, PRINTS, ProDom, SMART, ProSite, PANTHER), and the Cluster of Orthologous Groups of Proteins (COG) and KEGG databases were used to analyze the protein families and pathways. The DEPs were explored through GO, COG, and KEGG pathway analyses to determine their functional and biological properties, and a q-value ≤ 0.05 was defined as significantly enriched. The DEPs were used for volcanic map and cluster heatmap analyses.

### 3.5. Propidium Iodide (PI) and Fluorescein Diacetate (FDA) Staining of the Forl Mycelia

The *Forl* mycelia were treated with 0.8 mL/L of linalool for 12 h. Untreated mycelia were used as controls. After the treatment, the hyphae were collected, washed twice in PBS, and stained with 10 μM of PI (Solarbio Life Science, Beijing, China) and 10 μM of FDA (MackLin Biochemical, Shanghai, China) solution for 20 min, respectively. The stained hyphae were washed 3 times with PBS to remove any excess dye. The PI signal was measured at λex = 535/λem = 615 nm. The FDA signal was measured at λex = 490/λem = 520 nm using a laser confocal microscope (Eclipse 80i, Nikon, Tokyo, Japan). 

### 3.6. Determination of Cell Membrane Permeability

The conductivity of the mycelial suspension was measured using a portable conductivity meter (DDB-303A, Shanghai Yidian Scientific Instruments Co., Ltd., Shanghai, China) 0, 3, 6, 9, 12, 24, 48, and 72 h after the linalool treatment, respectively. The conductivity value at 0 h was marked J0, and that at 3–72 h was marked J1. The conductivity of the boiled mycelia after the final measurement was marked J2. The relative permeability rate of the mycelia was represented by the relative electric conductivity value, which was calculated as:

Relative electric conductivity (%) = [(J1 − J0)/(J2 − J0)] × 100(1)

### 3.7. Determination of Malondialdehyde (MDA) and Soluble Protein Contents

The MDA content was determined by the thiobarbituric acid (TBA) reaction, as described by Duan et al. [57]. In brief, mycelia were collected and homogenized in 0.1 M of PBS. The homogenate was centrifuged at 4000 rpm for 10 min, and 1 mL of the supernatant was mixed with 3 mL of 0.5% TBA (0.5 g of TBA, 20% trichloroacetic acid, 100 mL) and boiled at 95 °C for 30 min. After cooling, the OD values of the samples were measured at 450, 532, and 600 nm. MDA content was calculated using the formula:

MDA content (μmol/g) = [6.45 × (OD532 − OD600) − 0.56 × OD450] × V_t_/(V_0_ × W) (2)

where V_t_ (mL) is the volume of the total extract solution, V_0_ (mL) is the volume of the test solution, and W (g) is the fresh weight of the mycelia.

The soluble protein content of the samples was determined using the Bradford Coomassie Brilliant Blue (CBB) method [58]. A 15 μL aliquot of the protein extraction solution was mixed with 3 mL of CBB G-250, and the OD values of the samples were measured at 595 nm. The soluble protein content was calculated according to a standard curve with known protein concentrations. 

### 3.8. Determination of Superoxide (O_2_•^−^), Hydrogen Peroxide (H_2_O_2_), and Glutathione (GSH) Content, and Antioxidant Enzyme Activities

The H_2_O_2_, O_2_•^−^, and GSH contents were measured separately using assay kits (BC3590, BC1290, BC1170, respectively, Solarbio Life Science, Beijing, China), following the manufacturer’s instructions. The activities of catalase (CAT) (EC1.11.1.6), superoxide dismutase (SOD) (EC 1.12.1.11), and peroxidase (POD) (EC 1.11.1.7) were determined as described by Li et al. [59]. Each experiment was performed at least three times. 

### 3.9. Determination of Cell Wall Degradation-Related Enzyme Activity

The activity of the cell wall degradation enzymes, including chitinase, cellulase, and pectinase, was measured using commercial kits (BC2540, BC2540, BC2630, respectively, Solarbio Life Science, Beijing, China). After extraction, the suspension was centrifuged, and the activities were measured according to the manufacturer’s instructions. Each experiment was repeated at least three times.

### 3.10. Effects of Linalool in Controlling Root Rot Disease Caused by Forl

#### 3.10.1. Linalool Application and *Forl* Inoculation

The tomato cultivar “Provence” was used for all the experiments. The tomato plants were grown until 6 leaves developed, then they were pulled out of the soil. The tomato roots were washed with water and root dip-inoculated with 10 mL of spore suspension for 2 h, and then transplanted to sterile soil for another 15 days of growth. Sterile distilled water was used as the non-inoculated control. The evaluation of the antifungal potential of linalool was determined by soil drenching (approximately 50 mL per plant) after inoculating with *Forl*. 0.1% Tween80 was used as the non-treated control. The four treatments were the control (sterile distilled water combined with 0.1% Tween80), *Forl* (*Forl* inoculate combined with 0.1% Tween80), linalool (sterile distilled water combined with 0.8 mL of linalool), and linalool+

*Forl*: (*Forl* inoculate combined with 0.8 mL of linalool). Each tomato treatment consisted of 42 plants; the experiment was repeated three times. Plant weight and disease symptoms were assessed 15 days after the inoculation.

#### 3.10.2. Disease Severity Assessment

The disease index was scored as described by Gawehns et al. [60], according to the following scale: 0, no visible symptoms; 1, <30% of leaves yellowing, primary root slightly brown, normal lateral root; 2, >50% of leaves yellowing, primary root brown, lateral roots slightly brown; 3, wilting or dead, primary root very brown, lateral root disappears.

#### 3.10.3. Determination of Chlorophyll and Carotenoid Content

The chlorophyll (Chl *a* and Chl *b*) and carotenoid contents were measured to represent the physiological activity parameters. Then, 0.2 g of tomato leaves was extracted with 95% ethanol. The chlorophyll contents were estimated by measuring the absorbance at 649 and 665 nm. The absorbance of the carotenoids was detected at 480, 645, and 663 nm. Then, Chl *a*, *b*, and the carotenoid contents were calculated according to Ding et al. [61] and Lichtenthaler [62].

### 3.11. Plant Total RNA Extraction and Real-Time Quantitative PCR Analysis 

The tomato plants were inoculated as in 3.10.1, and leaf samples were collected at 1, 6, 12, 24, and 48 h after the linalool treatment, respectively. Non-inoculated plants and non-treated plants were used as control. Each tomato treatment consisted of 3 plants, and the experiment was repeated three times. Total RNA from the tomato plants was extracted using the AG RNAex Pro Reagent (Accurate Biotechnology Co., Ltd., Changsha, China), according to the manufacturer’s instructions. RNA was reverse-transcribed to cDNA using the HiScript II Q RT SuperMix for qPCR (+gDNA wiper) (Vazyme Biotech Co. Ltd., Nanjing, China). An RNA RT-qPCR was performed using the LightCycler^®^ 96 RealTime PCR System (Applied Biosystems, Foster City, CA, USA) with the SYBR^®^ qPCR Master Mix (Vazyme Biotech Co. Ltd., Nanjing, China). Gene expression was calculated using the 2^−ΔΔCt^ method [63]. *ACTIN* was selected as the control housekeeping gene. The RT-qPCR primers are listed in Table S12. 

### 3.12. Statistical Analysis

Data were analyzed using DPS software version 9.01, and the experimental results are expressed as mean ± standard deviation. The differences between the mean values were detected with Duncan’s multiple-range test. A *p*-value < 0.05 was considered significant.

## 4. Conclusions

Our results clearly validated the antifungal activity of linalool against the destructive soil-borne fungi *F. oxysporum f. sp. radicis-lycopersici* (*Forl*) in vitro and in vivo. The in vitro antimicrobial activity against *Forl* by linalool resulted from the structural disruption of cell membranes, redox dysfunction, and impeded nutrient metabolism. Linalool exhibited an excellent protective effect for tomato plants against *Forl* infection. It also increased the expression of the JA and SA pathways, which induce resistance to *Forl*. In addition to limiting the *Forl* infection, linalool promoted the growth of tomato plants. In summary, this study demonstrates that linalool is an effective substance to prevent *Forl* from damaging tomatoes. However, the effect of linalool on soil microorganisms, particularly the beneficial microbes, remains unclear, and requires further investigation before linalool is widely used in the field. 

## Figures and Tables

**Table 1:** List of DEPs related to metabolism, energy production and conversion, and pathogenicity.

Protein ID	Log_2_FC	*p*-Value	COG_Function_Description
Energy production and conversion			
FOXG_05055P0	1.50	0.0235	Isocitrate dehydrogenase
FOXG_14637P0	−1.36	0.0205	Malate/lactate dehydrogenase
FOXG_08948P0	−1.14	0.0140	FAD/FMN-containing dehydrogenase
FOXG_03411P0	1.56	0.0252	Acyl-CoA reductase or other NAD-dependent aldehyde dehydrogenase
FOXG_08681P0	−1.19	0.0128	Glycerol-3-phosphate dehydrogenase
FOXG_02187P0	−1.03	0.0129	Pyruvate/2-oxoglutarate dehydrogenase complex, dihydrolipoamide acyltransferase (E2) component
FOXG_13538P0	1.44	0.0134	Trans-aconitate methyltransferase
FOXG_02524P0	1.44	0.0041	FAD/FMN-containing dehydrogenase
Carbohydrate transport and metabolism			
FOXG_15373P0 **	−1.37	0.0050	Chitinase, GH18 family
FOXG_00102P0	−1.56	0.0205	*N*-acetyl-beta-hexosaminidase
FOXG_14504P0 **	−1.85	0.0018	Endo-1,4-beta-xylanase, GH35 family
FOXG_03723P0 **	−1.03	0.0224	Exo-beta-1,3-glucanase, GH17 family
FOXG_10748P0 **	−1.48	0.0019	Chitinase, GH18 family
FOXG_13407P0	−1.57	0.0268	6-phosphogluconolactonase, cycloisomerase 2 family
FOXG_02349P0 **	−1.18	0.0175	Periplasmic beta-glucosidase and related glycosidases
FOXG_05841P0	−1.47	0.0394	D-arabinose 1-dehydrogenase, Zn-dependent alcohol dehydrogenase family
FOXG_15329P0 **	−1.37	0.0028	Chitinase, GH18 family
FOXG_10052P0 **	−1.84	0.0010	Polygalacturonase
FOXG_10867P0	−1.39	0.0072	*N*-acetyl-beta-hexosaminidase
FOXG_05948P0 **	−1.76	0.0018	Pectate lyase
FOXG_03195P0	−1.47	0.0101	6-phosphogluconolactonase/Glucosamine-6-phosphate isomerase/deaminase
FOXG_06401P0	−1.03	0.0023	Mannose-6-phosphate isomerase, class I
FOXG_03963P0	−1.11	0.0266	6-phosphogluconolactonase, cycloisomerase 2 family
FOXG_07873P0 *	−1.20	0.0102	Na^+^/melibiose symporter or related transporter
FOXG_11081P0 **	−2.07	0.0462	Aryl-phospho-beta-D-glucosidase BglC, GH1 family
FOXG_03194P0 *	−1.04	0.0076	Hexokinase
FOXG_03843P0 *	−1.04	0.0278	Predicted arabinose efflux permease, MFS family
FOXG_08305P0	−1.79	0.0230	Predicted alpha-1,6-mannanase, GH76 family
FOXG_15351P0	−2.38	0.0087	Alpha-glucosidase, glycosyl hydrolase family GH31
FOXG_08602P0 **	−1.33	0.0233	Oxalate decarboxylase/archaeal phosphoglucose isomerase, cupin superfamily
FOXG_06388P0 *	−1.40	0.0065	Predicted arabinose efflux permease, MFS family
FOXG_10189P0	−1.45	0.0183	TPP-dependent 2-oxoacid decarboxylase, includes indolepyruvate decarboxylase
Lipid transport and metabolism			
FOXG_15474P0	−1.65	0.0188	Glycerophosphoryl diester phosphodiesterase
FOXG_12721P0	−1.11	0.0027	Acyl-CoA dehydrogenase related to the alkylation response protein AidB
FOXG_09686P0	−1.37	0.0057	Carboxylesterase type B
FOXG_13474P0	−1.29	0.0035	Carboxylesterase type B
FOXG_10416P0	−1.75	0.0017	Lysophospholipase, alpha-beta hydrolase superfamily
FOXG_11716P0	1.05	0.0022	Cyclopropane fatty-acyl-phospholipid synthase and related methyltransferases
FOXG_08523P0	1.09	0.0482	Fatty-acid desaturase
FOXG_11948P0	−1.36	0.0200	Lysophospholipase, alpha-beta hydrolase superfamily
FOXG_05822P0	−1.25	0.0011	Lysophospholipase, alpha-beta hydrolase superfamily
FOXG_12687P0	1.08	0.0109	Phosphatidate phosphatase APP1
FOXG_13507P0	2.35	0.0109	Acyl CoA:acetate/3-ketoacid CoA transferase, beta subunit
Pathogenicity-associated proteins			
FOXG_06095P0 *	−0.79	0.0098	Arylamine *N*-acetyltransferase 2
FOXG_01310P0 *	−0.53	0.0338	GTPase RHO3
FOXG_07946P0 *	−0.77	0.0424	GTPase-activating protein GYP7
FOXG_12808P0 *	−0.59	0.0145	GTP-binding protein ypt1
FOXG_13835P0 *	−0.70	0.0464	Small GTPase-binding protein
FOXG_01420P0 *	−0.56	0.0137	GTPase SAR1
FOXG_09867P0 *	−1.48	0.0024	Glutathione-dependent formaldehyde-activating enzyme
FOXG_06321P0 *	−0.56	0.0450	G-protein alpha subunit
FOXG_10547P0 *	−0.89	0.0111	Lipase ATG15

* Proteins associated with pathogenicity; ** cell wall-degrading enzymes.

## Data Availability

The data supporting the findings of this study are available within the article. The transcriptome raw data presented in the study are deposited in the NCBI Sequence Read Archive (SRA), and the accession number is PRJNA913446. The mass spectrometry proteomics data are available via ProteomeXchange with identifier PXD038942.

## Supplementary Materials

The following supporting information can be downloaded at: https://www.mdpi.com/article/10.3390/ijms24010458/s1.
