# Supplementary material for: Revealing the Mechanisms for Linalool Antifungal Activity against Fusarium oxysporum and Its Efficient Control of Fusarium Wilt in Tomato Plants
Source: Int J Mol Sci. 2022 Dec 27;24(1):458. doi: 10.3390/ijms24010458 (PMC9820380; doi:10.3390/ijms24010458)
Supplement: Supplementary file 1 [file ijms-24-00458-s001.zip › Supplementary figures.pdf]

International Journal of
Molecular Sciences

MDPI

Supplementary materials

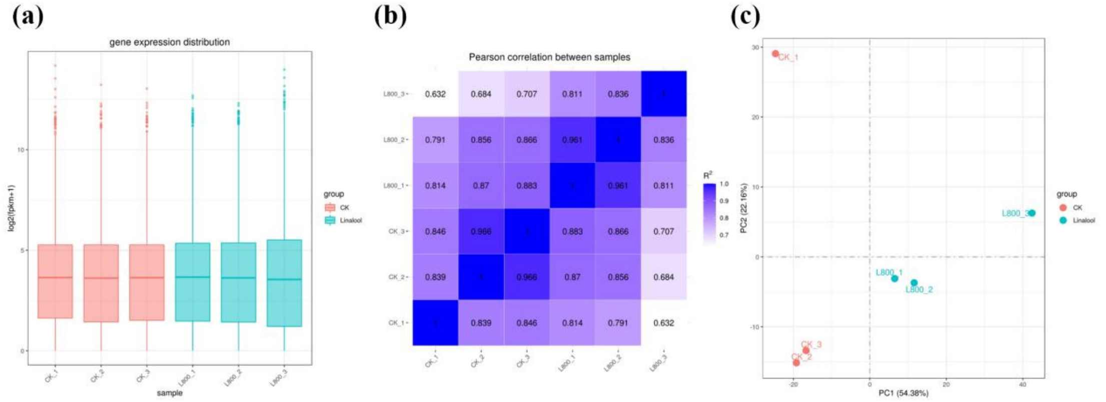

Figure S1. Expression distribution and relationship analysis between different transcriptomes. (**a**) Box plot showing the distribution of all TPMs in each sample. The ordinate is log2 (FPKM+1), and the horizontal line in the figure represents the median level of gene expression in the sample. (**b**) Principle Component Analysis (PCA) for each sample. (**c**) Heat map diagram of Pearson correlation coefficient between different samples.

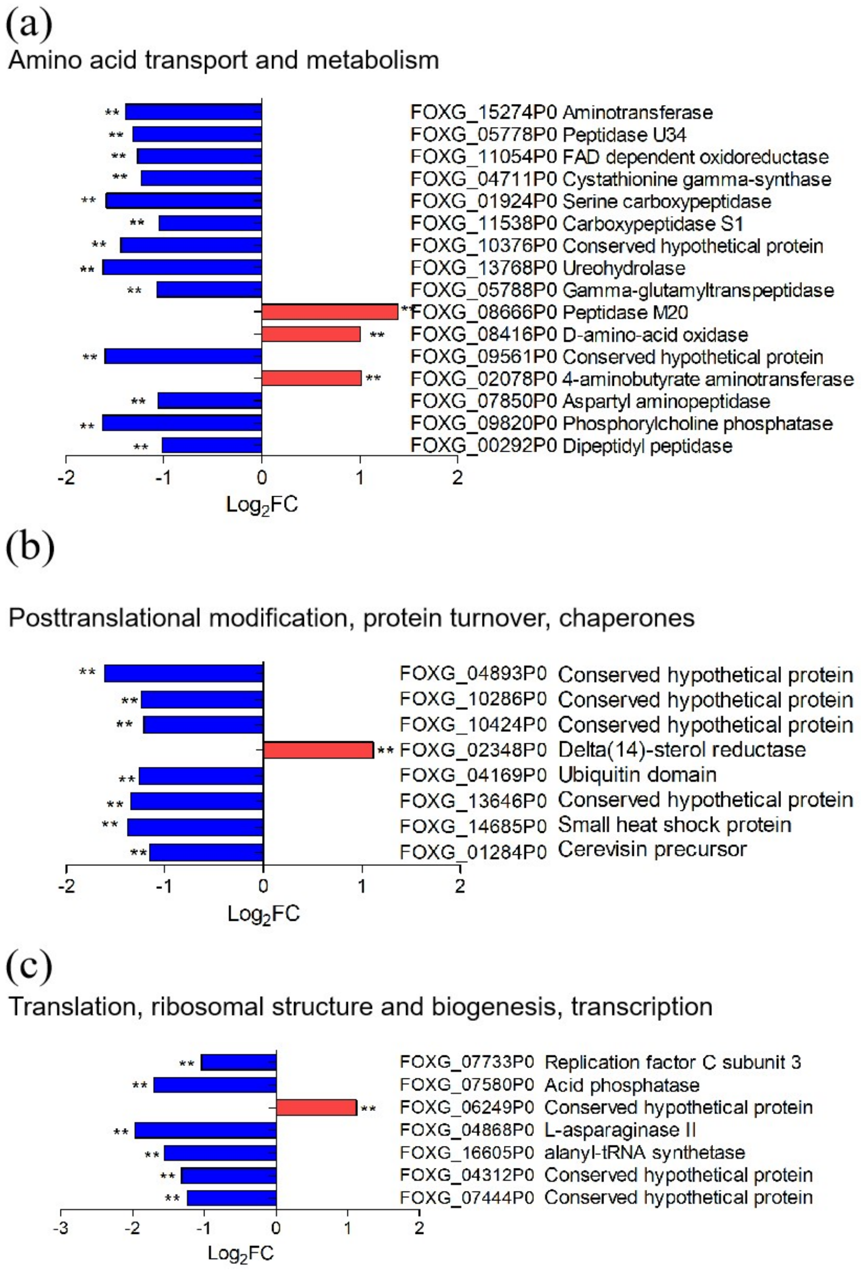

Figure S2. DEPs involved in amino acid transport and metabolism (**a**), posttranslational modification, protein turnover, chaperones (**b**), and translation, ribosomal structure and biogenesis, transcription (**c**) based on quantitative proteomic analysis. ∗∗represents $p < 0.05$.

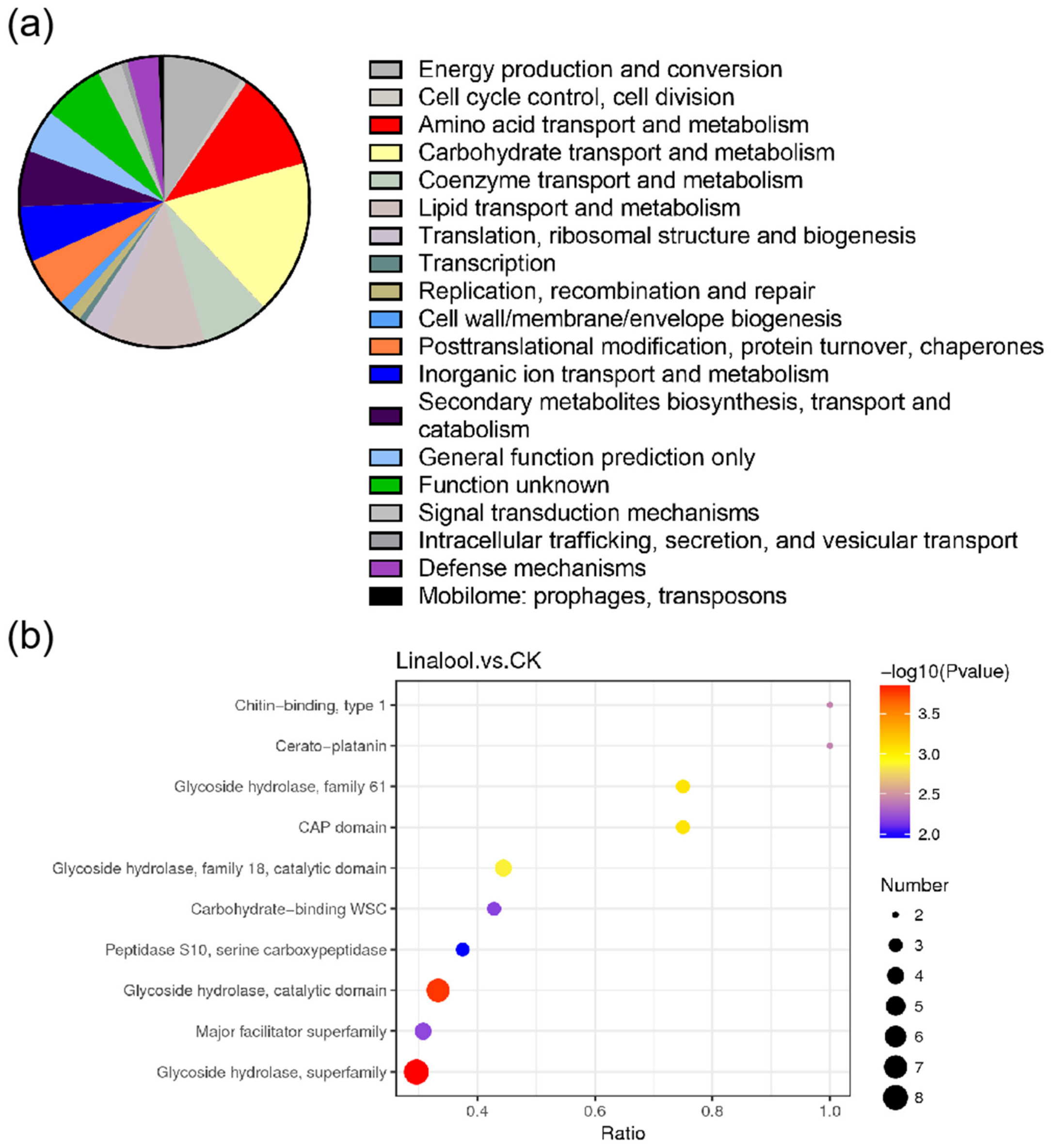

Figure S3. COG categories (**a**) and Interproscan (IPR) enrichment (**b**) of the DEPs.

Supplemental Tables

Supplemental Table S1 EC_{50} values (mL/L) of *Forl* on PDA media supplemented with the linalool evaluated.
Supplemental Table S2 Summary of RNA-Seq data generated for control and linalool treatment samples
Supplemental Table S3 The expression and annotations of all genes in linalool treatment and control samples
Supplemental Table S4 DEGs of *Forl* after 6 days of linalool treatment.
Supplemental Table S5 GO analysis of DEGs
Supplemental Table S6 KEGG analysis of EDGs
Supplemental Table S7 The expression and annotations of all proteins in linalool treatment and control samples
Supplemental Table S8 DEPs of *Forl* after 6 days of linalool treatment.
Supplemental Table S9 GO analysis of DEPs
Supplemental Table S10 KEGG analysis of DEPs
Supplemental Table S11 COG analysis of DEPs
Supplemental Table S12 Primers used for RT-qPCR.
